# The Efficacy of Bepotastine Besilate Compared With Hydroxyzine Pamoate for Preventing Infusion Reactions to the First Dose of Rituximab in Patients With Non-Hodgkin Lymphoma: Protocol for a Phase II, Double-Blind, Multicenter Randomized Trial

**DOI:** 10.2196/54882

**Published:** 2024-02-22

**Authors:** Yumi Kitahiro, Kazuhiro Yamamoto, Kimikazu Yakushijin, Takeshi Ioroi, Masaaki Tanda, Kotaro Itohara, Tomohiro Omura, Hironobu Minami, Ikuko Yano

**Affiliations:** 1 Department of Pharmacy Kobe University Hospital Kobe Japan; 2 Division of Medical Oncology/Hematology Department of Medicine Kobe University Hospital and Graduate School of Medicine Kobe Japan

**Keywords:** non-Hodgkin lymphoma, rituximab, infusion reactions, bepotastine besilate, histamine H_1_-receptor antagonist, hydroxyzine pamoate, drowsiness

## Abstract

**Background:**

Rituximab, an anti-CD20 monoclonal antibody, can cause infusion reactions (IRs), especially during the initial rituximab infusion therapy. Generally, patients are administered a histamine H_1_-receptor antagonist before the rituximab infusion, along with an antipyretic analgesic, to prevent or reduce IRs. Multiple retrospective case-control studies indicate that the second generation of histamine H_1_-receptor antagonists might be more effective than the first generation in suppressing IRs caused by the rituximab infusion.

**Objective:**

This study aimed to assess the efficacy of first- and second-generation histamine H_1_-receptor antagonists for preventing IRs resulting from the initial infusion of rituximab in patients diagnosed with non-Hodgkin lymphoma.

**Methods:**

This is a phase II, double-blind, active-controlled randomized trial. It will be a multicenter study conducted across 3 facilities that aims to enroll a total of 40 patients diagnosed with non-Hodgkin lymphoma who will receive their initial rituximab infusion. Participating patients will be administered hydroxyzine pamoate or bepotastine besilate, representing first- or second-generation histamine H_1_-receptor antagonists, respectively. This will be combined with 400-mg acetaminophen tablets taken approximately 30 minutes before the first infusion of rituximab. The primary end point of this trial is to assess severe IRs, equivalent to grade 2 or higher as defined by the National Cancer Institute Common Terminology Criteria for Adverse Events, version 5.0, that occur within a 4-hour period after the initiation of rituximab infusion. The secondary end points include assessing the severity of the initial IR, the maximum severity of the IR, and the duration between rituximab infusion initiation and the onset of the first IR within a 4-hour period. Additionally, the trial will evaluate histamine H_1_-receptor antagonist–induced drowsiness using the visual analogue scale, with each patient providing their individual response.

**Results:**

This study began with patient recruitment in April 2023, with 17 participants enrolled as of November 12, 2023. The anticipated study completion is set for February 2026.

**Conclusions:**

This study is the first randomized controlled trial comparing the effects of oral first- and second-generation histamine H_1_-receptor antagonists in preventing IRs induced by the initial administration of rituximab. The findings from this study hold the potential to establish the rationale for a phase III study aimed at determining the standard premedication protocol for rituximab infusion.

**Trial Registration:**

Japan Registry of Clinical Trials jRCTs051220169; https://jrct.niph.go.jp/latest-detail/jRCTs051220169

**International Registered Report Identifier (IRRID):**

DERR1-10.2196/54882

## Introduction

### Background

Rituximab, an anti-CD20 monoclonal antibody, is one of the most common chemotherapy agents used for non-Hodgkin lymphoma (NHL) [1-3]. Specifically, treatment protocols involving rituximab, cyclophosphamide, doxorubicin, vincristine, and prednisone (R-CHOP), as well as polatuzumab combined with rituximab, cyclophosphamide, doxorubicin, and prednisolone (Pola-R-CHP), have emerged as the established therapeutic standards for various NHL types, including diffuse large B-cell lymphoma [4,5]. In addition, a rituximab plus bendamustine (BR) regimen is a chemotherapy option for follicular lymphoma categorized as the NHL type [6].

Infusion reactions (IRs) commonly occur within the initial 24 hours of rituximab infusion and are a troublesome side effect associated with this treatment. Typically, the first rituximab infusion begins at a rate of 50 mg/h, increasing by 50 mg/h every 30 minutes, reaching a maximum of 400 mg/h [7]. Severe IRs tend to manifest 30 to 120 minutes after the initiation of rituximab infusion, especially during the escalation of the rituximab infusion rate [7]. The key symptoms include fever, pruritus, rash, and anaphylaxis-like symptoms, with reported instances of fatalities in severe cases [8,9]. While the precise mechanisms behind IRs remain unclear, the presence of tumor necrosis factor–α, interleukin-6, and other cytokines in the bloodstream at the time of administration might be responsible [10]. Moreover, immunoglobulin E–mediated type I reactions associated with histamine release have been identified as a potential mechanism for IR development in patients who previously experienced allergic reactions to rituximab [7,11]. Therefore, patients usually take an antipyretic analgesic and a histamine H_1_-receptor antagonist 30 minutes before rituximab infusion to mitigate or prevent an IR. However, as there is no standard premedication, the combination of an antipyretic analgesic and a histamine H_1_-receptor antagonist varies among medical facilities. Despite the frequent use of first-generation histamine H_1_-receptor antagonists [12,13], patients frequently experience IRs, particularly during the first rituximab infusion, even with these premedications [8]. In addition, first-generation histamine H_1_-receptor antagonists have potent central nervous system effects [14], causing drowsiness in patients both during and after rituximab infusion.

Nowadays, various retrospective case-control studies have indicated the potential superiority of the second generation of histamine H_1_-receptor antagonists over the first generation in suppressing the occurrence of IRs due to rituximab infusion [15,16]. However, these retrospective studies had a significant limitation, as they subjectively evaluated IRs based on individual physicians’ assessments. While a prospective study investigating the supplementary effects of second-generation drugs has been conducted, it specifically examined montelukast, a leukotriene receptor; rupatadine, a second-generation H_1_-receptor antagonist; or their combination alongside a standard premedication consisting of the first-generation H_1_-receptor antagonist diphenhydramine hydrochloride and acetaminophen [17]. Since the previous prospective study did not directly compare first-generation histamine H_1_-receptor antagonists with second-generation ones, its immediate applicability in clinical settings might be limited. Consequently, there is a need to establish an effective and safe premedication regimen that significantly suppresses IRs while causing minimal drowsiness.

Hence, we designed a prospective study to compare the impact of 2 antihistamines during the initial rituximab dose for NHL patients: hydroxyzine pamoate, a first-generation H_1_-receptor antagonist frequently used in clinical settings, and bepotastine besilate, a second-generation antihistamine identified in a retrospective case-control study as potentially superior in suppressing IRs triggered by rituximab infusion [15]. Hydroxyzine pamoate is superior to other first-generation H_1_-receptor antagonists such as chlorpheniramine and diphenhydramine, since it has no contraindications for angle-closure glaucoma or prostatic hyperplasia patients. It will be combined with acetaminophen to assess its effect on IR occurrence. This is an exploratory study, laying the groundwork by providing basic evidence for subsequent confirmatory studies.

### Study Objectives

The primary objective of this study is to estimate the incidence rate of IRs (grade 2 or higher) based on the National Cancer Institute Common Terminology Criteria for Adverse Events (CTCAE), version 5.0 [18], in each of the 2 study drug groups within the 4-hour period following the initiation of rituximab infusion. The secondary objectives include (1) estimating the severity of the first and the most severe IR within the same 4-hour time frame after rituximab infusion; (2) estimating the time of onset for the initial IR during this 4-hour period; and (3) estimating the rate of adverse events, including drowsiness attributed to histamine H_1_-receptor antagonists, using the visual analog scale (VAS).

## Methods

### Study Design and Study Location

**Figure 1:**
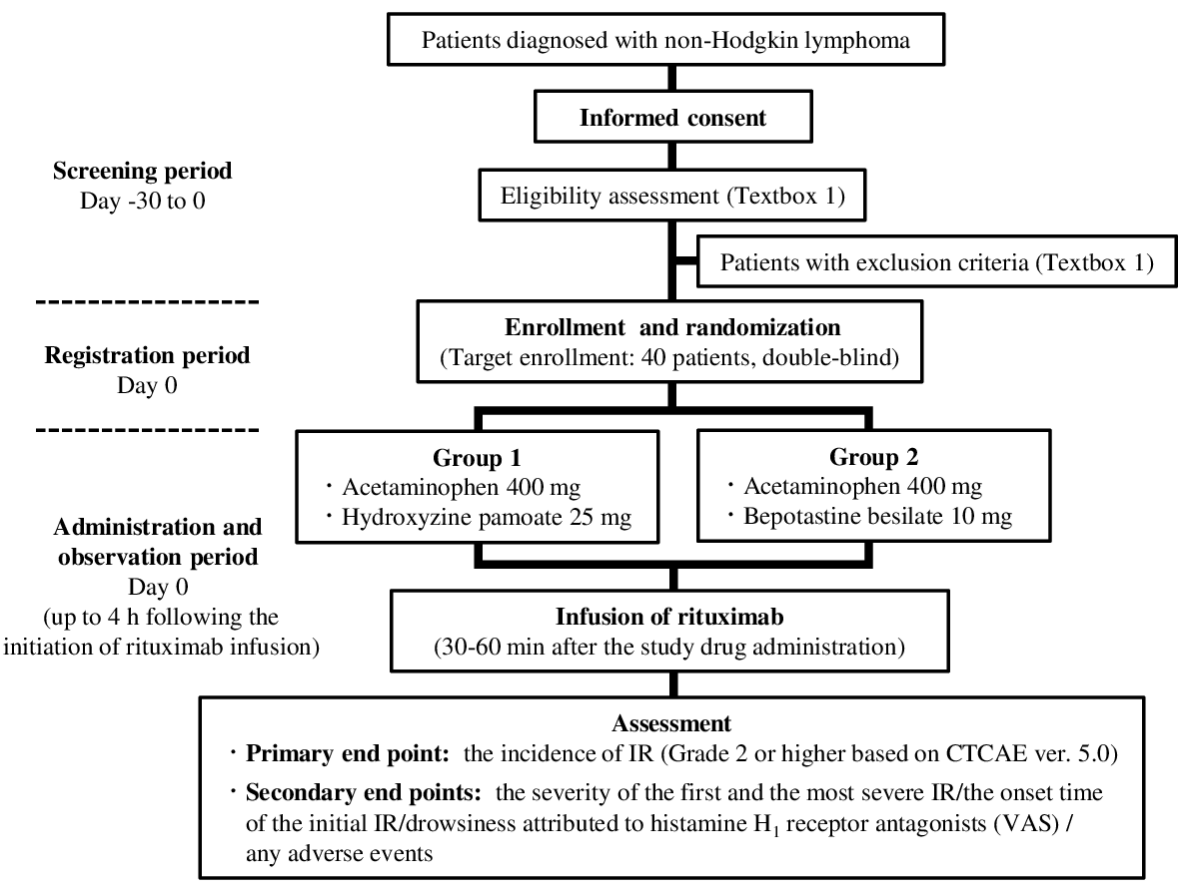
Flowchart illustrating the study design. CTCAE: National Cancer Institute Common Terminology Criteria for Adverse Events; IR: infusion reaction; VAS: visual analogue scale.

This study is an ongoing phase II, double-blind, active-controlled randomized trial (Figure 1). It is a multicenter study conducted across 3 facilities, including Kobe University Hospital.

### Ethical Considerations

This study was approved by the Kobe University Clinical Research Ethical Committee (C220009) on December 22, 2022. All participants will sign an informed consent form after receiving detailed explanations from the researchers. This study will adhere to the protocols and principles outlined in the Declaration of Helsinki. Any proposed changes to the protocol will require prior approval from the ethics committee before implementation.

### Inclusion and Exclusion Criteria

Textbox 1 outlines the criteria for inclusion and exclusion in this study. In addition, Table 1 shows the periods of restricted use of concomitant medication, which are listed in the exclusion criteria (Textbox 1; exclusion criteria 1-3). The specific time frames are determined by the half-life of each medication.

**Study inclusion and exclusion criteria.**

**Inclusion criteria**

1. Aged 18 years or older at the time of consent
2. Written informed consent and voluntary participation in this clinical study
3. Diagnosed with non-Hodgkin lymphoma
4. Receiving a first rituximab infusion, irrespective of regimen
5. Receiving rituximab as a standalone treatment before other anticancer agents in one of the following regimens: rituximab, cyclophosphamide, doxorubicin, vincristine, and prednisone (R-CHOP); polatuzumab combined with rituximab, cyclophosphamide, doxorubicin, and prednisolone (Pola-R-CHP); and rituximab plus bendamustine (BR)

**Exclusion criteria**

1. Administered or scheduled to receive the following medications orally or intravenously within 2 days of the study drug administration: a short half-life antipyretic analgesic (listed in Table 1) or corticosteroid
2. Administered or scheduled to receive histamine H_1_-receptor antagonists orally or intravenously within 5 days of the study drug administration
3. Administered or scheduled to receive a long half-life antipyretic analgesic (listed in Table 1) orally or intravenously within 10 days of the study drug administration
4. Received obinutuzumab before the rituximab treatment
5. Presence of renal dysfunction, indicated by a creatinine clearance <50 mL/min
6. Presence of liver dysfunction, indicated by a Child-Pugh score of C
7. Severe interstitial pneumonia
8. Porphyria
9. Pregnancy or possible pregnancy
10. Known allergies or sensitivities to the drugs used in this clinical study
11. Determined to be unsuitable for inclusion by the investigator

**Table 1:** Prohibited period of concomitant use of medications as part of the exclusion criteria.

Prohibited period before study drug administration	Category and nonproprietary name
10 days	Antipyretic analgesics with a long half-life (oxaprozin, piroxicam, meloxicam, nabumetone, sulindac, and naproxen)
5 days	All histamine H_1_-receptor antagonists
2 days	Antipyretic analgesics with a short half-life (acetaminophen, etodolac, celecoxib, flurbiprofen axetil, pranoprofen, flurbiprofen, lornoxicam, ibuprofen, tiaprofenic acid, ketoprofen, indometacin, loxoprofen sodium hydrate, diclofenac sodium, and acetylsalicylic acid), all corticosteroids

### Study Drugs

The study drugs are prepared at the Department of Pharmacy, Kobe University Hospital. To maintain the blind nature of the study, the drug preparation involves placing either a 25-mg hydroxyzine pamoate capsule or a 10-mg bepotastine besilate tablet inside an opaque capsule (size 2).

### Intervention

Patients are randomly allocated to receive either hydroxyzine pamoate or bepotastine besilate based on the presence or absence of bone marrow infiltration [19]. Approximately 30 minutes before the rituximab infusion, patients will take a combination of the assigned H_1_-receptor antagonist with acetaminophen tablets (400 mg). To maintain the blind nature of the study, the block size used for randomization will remain undisclosed and concealed until the completion of all analyses. Throughout the study’s duration, the randomization information will be kept confidential by the data management and statistical analysis team. The allocation manager, independent of the study, will retain the randomization details. The study schedule is outlined in Table 2. Rituximab administration follows the regimen specific to each facility and is delivered intravenously. In general, the first infusion starts at an initial rate of 50 mg/h, escalating every 30 minutes by increments of 50 mg/h to a maximum of 400 mg/h [17]. All rituximab products (original or biosimilar) are allowed in each facility and the information on the rituximab administration schedule will be recorded.

**Table 2:** Summary of the study schedule.

	Screening period (day –30 to 0)	Registration period (day 0)	Administration and observation period (day 0)
Informed consent	✓		
Eligibility screening	✓		
Registration		✓	
Randomization		✓	
Patients’ backgrounds^a^	✓	✓	
Ann Arbor classification	✓		
Eastern Cooperative Oncology Group Performance Status Scale	✓		
Child-Pugh score	✓		
Stratification factors (the presence or absence of bone marrow involvement)	✓		
Hematology^b^ and biochemistry^c^ tests	✓		
B symptoms (the presence or absence of fever, night sweat, or weight loss)	✓		
Administration of study drug			✓
Evaluation of infusion reaction			✓
Observation of adverse events			✓
Evaluation of drowsiness (visual analogue scale; performed 90, SD 15 minutes after study drug administration)			✓

^a^Including age, sex, weight, height, diagnosis, medical history, complications, and concomitant medications.

^b^Hematology tests encompass red blood cell counts, hemoglobin, hematocrit, differential leukocyte counts, and platelet counts.

^c^Biochemistry tests include aspartate aminotransferase, alanine aminotransferase, γ-glutamyl transpeptidase, total bilirubin, albumin, creatinine, blood urea nitrogen, lactate dehydrogenase, IL-2 receptor, and prothrombin activity.

### Data Collection and Management

Upon receiving consent from prospective patients, the investigators assess their eligibility. Subsequently, either the research secretariat or the branch have access to the REDCap (Research Electronic Data Capture; an electronic data system for clinical research) system to confirm eligibility before inputting essential information for case registration and assigning a case number. The registration process concludes upon the display of a confirmation of the case registration.

### Study Outcomes

#### Primary End Point

The primary end point of this study is the incidence of IR (grade 2 or higher) according to CTCAE within the time frame of up to 4 hours following the initiation of rituximab infusion.

#### Secondary End Points

The secondary end points of this study include assessing the severity of the initial IR, the maximum severity of the IR, the duration between rituximab infusion initiation and the onset of the first IR within a 4-hour period, evaluating drowsiness due to histamine H_1_-receptor antagonists, and determining the incidence rate of any adverse events resulting from participation in clinical research.

### Assessments

#### Evaluation of IR

Investigators assess the severity of the IR using the CTCAE (Table 3) as a reference. Furthermore, they evaluate IR severity based on the specific criteria outlined in this study, aligned with routine medical care. Grade 1 is a temporary interruption of rituximab infusion followed by a restart at the same rate. Grade 2 or higher necessitates a temporary halt in infusion along with interventions such as additional symptom treatment or resumption of infusion at a reduced rate. Grade 2 or higher also includes situations where the rituximab infusion rate remains unchanged due to concerns about the patient’s condition. In the event of an IR, standard rescue care protocols specified by the medical facility are implemented. Procedures for rituximab dosing rates and rate adjustments are also implemented.

**Table 3:** The Common Terminology Criteria for Adverse Events, version 5.0, defines infusion-related reaction. The items in the Criteria column are extracted directly from the source [18].

Grade	Criteria
1	Mild transient reaction; infusion interruption not indicated; intervention not indicated
2	Therapy or infusion interruption indicated but responds promptly to symptomatic treatment (e.g., antihistamines, NSAIDs, narcotics, intravenous injection fluids); prophylactic medications indicated for < = 24 h
3	Prolonged (e.g., not rapidly responsive to symptomatic medication and/or brief interruption of infusion); recurrence of symptoms following initial improvement; hospitalization indicated for clinical sequelae
4	Life-threatening consequences; urgent intervention indicated
5	Death

#### Evaluation of Drowsiness Caused by Histamine H_1_-Receptor Antagonists

Patients report the severity of drowsiness using a VAS ranging from 0 mm (awake) to 100 mm (falling asleep) approximately 90 (SD 15) minutes after taking the study medication.

### Statistical Procedure

All analyses will be carried out using R (R Foundation for Statistical Computing). There will be no interim analyses conducted.

### Sample Size Calculation

The target sample size for this study is 40 participants, evenly divided into groups receiving hydroxyzine pamoate and bepotastine besilate, with 20 patients in each group. The main objective is to compare the incidence of IR (grade 2 or higher) according to the CTCAE within 4 hours after initiating rituximab infusion in the 2 study drug groups. In our retrospective exploratory study at Kobe University Hospital, grade 2 or higher IR incidence was 8/30 (27%) in NHL patients who received hydroxyzine pamoate before their first rituximab dose. In contrast, a previous report indicated that the incidence was 3/33 (9%) in patients treated with bepotastine besilate [15]. Therefore, we conservatively estimated that the grade 2 or higher IR incidence would be 25% for hydroxyzine pamoate and 10% for bepotastine besilate. This assumption implies a 15% difference in the IR incidence rate between the 2 groups. Assuming 20 patients in each group, the calculated 90% and 95% CIs of the incidence difference between the 2 groups are –0.044 to 0.344 (width 38.8%) and –0.081 to 0.381(width 46.2%), respectively. If both widths for the 90% and 95% CIs of the incidence difference of observed data are narrower than the presumed interval widths, respectively, we consider the accuracy of the estimates suitable for planning a phase III trial.

### Primary Analysis

In this study, a full analysis set (FAS) comprises patients randomly assigned to the study who completed the evaluation of IR within 4 hours after rituximab infusion initiation. We will analyze the difference in IR incidence between the 2 study drug groups within the FAS and determine their respective 90% and 95% CIs. The calculation of Clopper-Pearson CIs will be used for this analysis.

### Secondary Analysis

A supplementary assessment will be conducted using the per-protocol set (PPS) for the primary outcome. In this study, the PPS consists of patients excluded from the FAS due to significant violations, such as incompatibility in meeting the selection or exclusion criteria and severe noncompliance with the research protocol. For each analysis, the null hypothesis will be that the IR incidence rate is equal between the 2 groups. Following this, we will calculate the *P* value using the Pearson chi-square test. The analysis of secondary outcomes aims to provide additional insights into the primary outcome. The severity of the initial and the maximum severity of IR will be indicated by the number and percentage within each grade. The null hypothesis for each analysis population will be that the IR incidence rate is equal to each grade within the 2 groups. The *P* value will be calculated using the Fisher exact test. The time to the onset of the initial IR and the VAS value indicate drowsiness caused by histamine H_1_-receptor antagonists. These will be presented as the mean (SD) for each group, along with the 95% CI for the mean difference.

### Data Monitoring and Pharmacovigilance

The study will undergo regular monitoring to safeguard human rights and welfare. The research will be conducted in strict adherence to the protocol and all relevant regulatory requirements, ensuring safety. The principal investigator has designated responsible individuals to oversee the outlined procedures for study monitoring. To ensure quality control, the monitor will assess adherence to the protocol and outlined procedures during the study.

In this research, an adverse event encompasses any illness, disability, infection, or death occurring during the study specifically related to the study drugs but excluding those related to rituximab and acetaminophen. Investigators will document these adverse events in the electronic case report form (eCRF). Affected patients will receive appropriate treatment and continued monitoring until symptoms resolve, as managed by the investigators. If there are cases in which the investigators identify unpredictable adverse events or deaths potentially linked to the study drug, these occurrences will be promptly reported to the review board. To address potential claims resulting from health issues related to the study’s conduct, the principal investigator has secured clinical research insurance covering compensation for death, serious disability, medical expenses, and medical benefits.

### Privacy and Confidentiality

The eCRF will be protected by using a password. Additionally, privacy measures will involve using deidentified data instead of personal identifiers across all eCRF entries to further enhance security.

## Results

This study is ongoing, and recruitment of participants began in April 2023, with 17 patients enrolled as of November 12, 2023. Anticipated study completion is scheduled for February 2026. The study protocol and statistical analysis plan will be made available on the Japan Registry of Clinical Trials. The study findings will be presented at medical conferences and published in scientific papers. Subsequent to publication, the corresponding author will make the collected data accessible in a non–personally identifiable form on reasonable request from other researchers within a prescribed period.

## Discussion

### Principal Findings

This phase II study is designed as a double-blind, prospective investigation, building upon insights from our retrospective exploratory study at Kobe University Hospital and a previous retrospective study [15]. While various reports have discussed IR prophylaxis during rituximab infusion, most have primarily assessed the efficacy of first-generation histamine H_1_-receptor antagonists. Recent retrospective case-control studies have suggested the potential superiority of second-generation histamine H_1_-receptor antagonists in suppressing IRs triggered by rituximab infusion. However, these studies often carried significant limitations, as IR assessments were subjective and varied among physicians [15,16]. This prospective study aims to address the research bias commonly found in retrospective studies and aims to provide more reliable results.

Severe IRs typically tend to manifest between 30 to 120 minutes after the commencement of rituximab infusion, as well as when the rituximab infusion rate is escalated [7]. Given that our study involves both inpatients and outpatients, assessing IRs over an entire day becomes challenging as outpatients leave the outpatient chemotherapy room after the rituximab infusion. To streamline the evaluation process without having the assessments focus only on inpatients, we considered a practical time frame for evaluation. Our retrospective exploratory study showed that the total rituximab infusion time was approximately 4 hours. Therefore, we have set the evaluation period for IR up to 4 hours following the initiation of rituximab infusion to align with actual practice.

It has been reported that the time to maximum concentration (T_max_) of histamine H_1_-receptor antagonists when used as premedication can impact prophylaxis against an IR [15]. In our retrospective exploratory study at Kobe University Hospital, we observed that the incidence time of IR was a median 90 (IQR 60-135) minutes after the onset of rituximab infusion in NHL patients who had received hydroxyzine pamoate before their initial rituximab dose. The reported mean T_max_ values for hydroxyzine pamoate and bepotastine besilate are 2.1 (SD 0.4) hours [20] and 1.2 (SD 0.2) hours [21], respectively. Notably, when patients take the H_1_-receptor antagonist approximately 30 minutes before rituximab infusion, bepotastine besilate reaches T_max_ before the median IR incidence time. To maintain blinding in this study, the study drug is encapsulated in an opaque gelatin capsule. Considering that the mean disintegration time for these capsules in the fed state was reported to be 12 (SD 4) minutes [22], we do not anticipate that the use of capsules will influence the study results. However, it is unclear whether the T_max_ of histamine H_1_-receptor antagonists only impacts prophylaxis against IRs. Among first-generation H_1_-receptor antagonists used for rituximab premedication in a clinical setting, the T_max_ of hydroxyzine pamoate is closest to bepotastine. We assume that class effects other than T_max_ contribute to the prevention of IRs. Furthermore, it is essential to consider that drowsiness resulting from histamine H_1_-receptor antagonists is influenced not only by their half-lives but also by their interaction with H_1_ receptors in the central nervous system [23,24]. These factors will be taken into consideration during the final assessment.

Several studies have investigated the correlation between the development of an IR and various risk factors. Factors such as soluble interleukin-2 receptor, hemoglobin, bone marrow infiltration, and lactate dehydrogenase levels have been identified as potential risk factors for IRs [19,25,26]. In this study, bone marrow infiltration is being treated as a stratification factor. We will also explore the potential associations between IR occurrence and multiple factors in an exploratory manner. However, due to the inclusion of a small and limited number of patients, our study serves as a preliminary investigation to determine whether further confirmation studies are warranted.

If this study demonstrates increased effectiveness of bepotastine besilate, a second-generation histamine H_1_-receptor antagonist, a confirmatory study will be designed to gather robust evidence supporting its effective use in routine practice.

### Conclusions

This study is the first randomized controlled trial comparing the effects of oral first- and second-generation histamine H_1_-receptor antagonists in preventing IRs induced by the initial administration of rituximab. The findings from this study have the potential to establish a rationale for a phase III study aimed at determining a standard premedication protocol for rituximab infusion.

## Abbreviations

BR: bendamustine hydrochloride plus rituximab
CTCAE: Common Terminology Criteria for Adverse Events
eCRF: electronic case report form
FAS: full analysis set
IR: infusion reaction
NHL: non-Hodgkin lymphoma
Pola-R-CHP: polatuzumab plus rituximab, cyclophosphamide hydrate, doxorubicin hydrochloride, and prednisolone
PPS: per-protocol set
R-CHOP: rituximab plus cyclophosphamide hydrate, doxorubicin hydrochloride, vincristine sulfate, and prednisolone
REDCap: Research Electronic Data Capture
Tmax: time to maximum concentration
VAS: visual analogue scale

## Data Availability

The data sets generated or analyzed during this study are available from the corresponding author on reasonable request.
